# Latitudinal and Longitudinal Trends of Seed Traits Indicate Adaptive Strategies of an Invasive Plant

**DOI:** 10.3389/fpls.2021.657813

**Published:** 2021-06-10

**Authors:** Lifeng Zhou, Hongwei Yu, Kaiwen Yang, Li Chen, Wandong Yin, Jianqing Ding

**Affiliations:** State Key Laboratory of Crop Stress Adaptation and Improvement, School of Life Sciences, Henan University, Kaifeng, China

**Keywords:** latitude, longitude, climate, germination, *Ambrosia artemisiifolia*, invasion

## Abstract

Invasive plants may change their seed traits to adapt to the environment and facilitate their performance. Studies on variation in seed traits among populations of an invader along latitudes/longitudes may assist in revealing how invasive plants cope with variable climates. In this study, we collected seeds of 26 populations of the global invasive plant *Ambrosia artemisiifolia* along ranges spanning 23° latitudes and 20° longitudes that are highly correlated in its invasive range in China. We measured over 20 seed traits, including seed morphology, phytohormone, nutrients, and germination, and investigated how the climate along the latitudes affects those traits. We found that germination time was significantly delayed with increasing latitude and longitude, while the reversed patterns were true for the germination rate. From low to high latitude, seed size, abscisic acid, and fatty acid were increased, likely affecting seed germination. Our analysis further demonstrated that temperature is the dominant driver of the variability in seed traits and germination. Germination rates of larger seeds in cold ranges were lower, while smaller seeds from warm ranges germinated faster, likely indicating adaptive strategies of the invasive plant in seed trait functional ecology. Together, our findings provide new insights into understanding the seed adaptation strategies during the invasion process and the underlying physiological and biochemical mechanisms involved.

## Introduction

Many invasive plants reproduce *via* seeds; thus seed traits are critical for invasive plant performance during their invasion process. Numerous studies have indicated that plant traits such as biomass, growth rate, and competitive ability are largely related to their successful invasion (van Kleunen et al., 2010; Divíšek et al., 2018). However, it remains unclear how invasive plants change their seed traits to adapt to environments and facilitate their performance. For seed traits, several previous studies have focused on the difference in germination traits of an invasive species between its native and introduced regions (Xu et al., 2019; Zhang et al., 2019). Further studies on the variation in seed traits among introduced populations of an invader may assist to explicitly reveal how the invader copes with variable environments. Moreover, examining differences in physiological and biochemical traits of seeds among populations of invasive plants could also provide insights for understanding seed trait functional ecology (Saatkamp et al., 2019). Such knowledge can ultimately deepen our understanding of species invasion mechanisms and improve our ability to predict future plant distribution and expansion.

Variations in seed traits such as seed size, mass, and morphology between populations of a plant species could be driven by geographical or environmental variables, including temperature and precipitation (Soper Gorden et al., 2016; Jesús et al., 2017; Mojzes et al., 2018). For example, several studies have revealed that there is a negative relationship between seed mass/size and latitude and plants produce larger/heavier seeds in lower latitudes with higher temperatures or greater rainfalls within species (Jesús et al., 2017; Wu et al., 2018). Conversely, Graae et al. (2009) found that seed mass increased significantly by latitude within species. Similarly, another study found two *Acer* spp. increased seed size at higher latitudes (Carón et al., 2014). A recent study also identified that seed size is associated with climate factors over large geographic areas, and the effect differs between species (Soper Gorden et al., 2016). Many studies reported invasive plant growth adaptation along latitudinal or temperature gradients (Clevering et al., 2001; Kollmann and Bañuelos, 2004; Liu et al., 2020) and variations in reproductive characteristics (Moravcova et al., 2010; Moravcová et al., 2015). However, the latitudinal and/or longitudinal trends of invasive plant seed traits remain poorly understood. Hence, revealing adaptative strategies of invasive plant seed traits by examining their variation among populations in different latitudes/longitudes is important to understand the effects of geographic and environmental factors on seeds.

Germination is one of the key seed traits, determining the establishment and expansion of plant populations (Donohue et al., 2010; Molina-Montenegro et al., 2018; Saatkamp et al., 2019). Studies indicate that the geographic variation in seed germination depends on climatic variables (Soper Gorden et al., 2016; Wu et al., 2018). For example, relative to populations in low latitudes, the germination time of populations of *Erica* spp. in high latitudes was delayed (Chamorro et al., 2018), indicating the effects of the local climate along latitudes. Such variation in germination timing could immediately and substantially affect the seedling stage, the most vulnerable period in the life cycle (Leiblein-Wild et al., 2014; Ortmans et al., 2016). Particularly for widespread invasive species, successful germination may increase their competitive advantages for the establishment and population recruitment in new environmental conditions (Wainwright and Cleland, 2013). However, since resources are limited, trade-offs between seed traits of invasive plants may exist. Therefore, as a response to various environmental conditions along latitudinal and longitudinal gradients, an invasive plant may adapt different strategies in germination, leading to intraspecific variation in seed traits (Leiblein-Wild and Tackenberg, 2014; Wu et al., 2018). For example, high latitudes with harsh conditions (e.g., lower temperature and precipitation) may drive plants of an invader increase their tolerance by producing larger seeds relative to those in low latitudes. Conversely, low latitudes with suitable climates may stimulate population enhancing their competitiveness by germinating earlier with smaller seeds. These predictions, which are important to understanding plant invasion success and predicting range extension responding to future climate change, however, have never been tested.

Understanding seed traits affecting germination is essential when investigating variation in germination. Seed morphological traits, including seed size and seed mass, have been found to influence the rate and speed of germination (Baskin and Baskin, 2014). Most studies have demonstrated that larger/heavier seeds demonstrate higher germination rate due to more resource reserves (Hantsch et al., 2013; Li et al., 2015; Wu et al., 2018). However, Pivatto et al. (2014) found that seed mass had no significant relationship between them, and Bu et al. (2007) found a significant negative correlation between seed mass and germinability based on a germination test database with 558 species. Seed chemical traits, such as phytohormone, structural material, and seed nutrients, also play important roles in seed germination. Abscisic acid (ABA) positively regulates dormancy and negatively affects germination, while gibberellins (GA) release dormancy and promote germination, counteracting ABA effects (Kucera et al., 2005; Miransari and Smith, 2014). Indoleacetic acid (IAA) may also strictly regulate seed dormancy alongside ABA (Miransari and Smith, 2014; Shu et al., 2016). Zhao et al. (2018) found that soluble sugar was significantly positively correlated with the germination rate, but there was no effect regarding starch content. Moreover, fatty acids are the major reserves mobilized during germination and early seedling growth (Erbas et al., 2016). For the invasive plant *Triadica sebifera*, Zhang et al. (2019) found that seeds from the introduced range germinated faster than those from the native range. Additionally, seeds from the introduced range were larger, with higher GA concentrations and a higher GA: ABA ratio, but lower crude fat concentrations compared to those from the native range. However, to date, there are no studies reporting the patterns of biochemical seed traits of invasive plants along latitudes.

Here, we report the potential adaptive strategies of germination of the invasive plant, *Ambrosia artemisiifolia* (common ragweed), by examining the driving factors of seed traits along latitudes/longitudes in China, one of the introduced ranges of this worldwide invader. Previous studies have demonstrated that this plant generally produces heavier seeds in higher latitudes in Australia (van Boheemen et al., 2019), and larger seeds produce a higher above-ground biomass of seedlings (Ortmans et al., 2016), thereby increasing their frost tolerance (Leiblein-Wild et al., 2014). Furthermore, within a certain range, higher temperature facilitates this plant germination process (Wang et al., 1999; Sang et al., 2011; Farooq et al., 2019). However, there is a lack of information of the plant’s adaptive strategies of gemination trends along latitudes.

In this study, we collected seeds of *A. artemisiifolia* from 26 populations along a latitudinal gradient in China and measured 24 seed traits (four germination and 20 morphological and chemical traits). We investigated the relationships between seed traits and latitude/longitude, and explored how seed morphological and chemical attributes affect seed germination traits. We further explored how the climate along various latitudes/longitudes directly and indirectly affect seed germination. We predict that the populations along latitudes may have different strategies in germination. Specifically, we address the following questions: (i) Are there notable variations in seed traits (mass, germination, nutrients, and biochemical property) among populations? (ii) If so, are there trends along latitudinal and longitudinal gradients? (iii) What are the major environmental drivers underlying the variation in seed traits for such trends?

## Materials and Methods

### Study Species and Sample Collection

*Ambrosia artemisiifolia* L. (common ragweed, Asteraceae), native to North America, is an annual plant propagated by seeds, is self-incompatible and wind pollinated. Since its introduction into China in the 1930s, this species is now widely distributed across the country geographically from 23° N to 46° N latitude (Wan et al., 2009). In the present study, seeds of 26 populations were collected at different locations in China (23.45–44.64°N, 103.94–129.70°E; Table 1 and Supplementary Figure 1). A previous study indicated *A. artemisiifolia* populations in China was introduced multiple times from different source regions (Li et al., 2019). According to their genetic background, we chosen populations sharing a low level of genetic differentiation for our study. In the present study, seeds of 26 populations were collected from late October to early December in 2018 (Table 1) according to their maturity along with latitudinal gradient. For each population, we collected seeds from 10–15 plants that were at least 10 m apart from each other. In each population, undamaged and mature seeds from approximately 3 plants were pooled together as a sample. There were 3–5 samples per population and 119 samples in total (Table 1). Seeds were air dried, followed by storing at 4°C with a desiccant for 12 weeks to break dormancy (Willemsen and Rice, 1972).

**TABLE 1:** Geographic locations and home climate (mean annual temperature, MAT; mean annual precipitation, MAP) of 26 populations of *Ambrosia artemisiifolia* used in this study.

**Pop.**	**Latitude (°N)**	**Longitude (°E)**	**MAT (°C)**	**MAP (mm)**	**Collection location**
BDG	44.64	129.66	4.62	610.86	Mudanjiang, Heilongjiang
BS	44.61	129.59	4.62	610.86	Mudanjiang, Heilongjiang
AH	44.58	129.70	4.62	610.86	Mudanjiang, Heilongjiang
KLT	44.56	129.56	4.62	610.86	Mudanjiang, Heilongjiang
FJC	44.53	129.58	4.62	610.86	Mudanjiang, Heilongjiang
SJC	41.97	123.83	7.00	775.22	Shenyang, Liaoning
QPS	41.95	123.64	7.00	775.22	Shenyang, Liaoning
LDC	41.92	123.79	7.00	775.22	Shenyang, Liaoning
HY	39.96	119.56	10.39	665.88	Qinhuangdao, Hebei
CGZ	39.87	119.47	10.39	665.88	Qinhuangdao, Hebei
GD	39.78	119.31	10.39	665.88	Qinhuangdao, Hebei
YC	32.77	117.99	16.24	1,106.70	Chuzhou, Anhui
MAZ	32.29	118.59	15.80	1,171.52	Chuzhou, Anhui
HW	32.15	118.46	15.80	1,171.52	Chuzhou, Anhui
TQ	29.43	113.45	17.95	1,382.38	Yueyang, Hunan
PS	29.38	113.42	17.95	1,382.38	Yueyang, Hunan
MT	29.24	115.63	17.32	1,760.53	Jiujiang, Jiangxi
TL	29.19	115.47	17.32	1,760.53	Jiujiang, Jiangxi
AC	29.09	115.77	17.32	1,760.53	Jiujiang, Jiangxi
DJ	28.95	113.26	17.95	1,382.38	Yueyang, Hunan
DX	25.02	113.72	20.35	1,544.10	Qingyuan, Guangdong
JT	24.00	109.08	20.31	1,770.27	Qingyuan, Guangdong
SJ	23.67	109.53	21.68	1,757.08	Qingyuan, Guangdong
MC	23.60	109.37	21.52	1,553.00	Laibin, Guangxi
ST	23.48	103.94	21.52	1,553.00	Laibin, Guangxi
SY	23.45	109.44	21.52	1,553.00	Laibin, Guangxi

*Climate variables were derived from 19-year (2000–2018) averages and downloaded from the China Meteorological Data Service Center web page.*

### Morphological Traits

Images of ten seeds randomly selected from each sample were captured with a high-speed scanner, and the size was analyzed by ImageJ software and averaged to calculate the seed length and width. Fresh mass (W_1_) of 100 seeds for each group were weighed, and the dry mass (W_2_) was recorded post oven-dried at 60°C for 72 h. Moisture content (%) was calculated as [(W_1_–W_2_)/W_1_] × 100.

### Chemical Traits

A total of 119 samples from 26 populations were used for chemical traits experiments. Phytohormone levels of fresh seeds, including GA, IAA, and ABA, were measured using enzyme-linked immunosorbent assay kits (ELISA) (Shanghai Enzyme-linked Biotechnology Co., Ltd.), following the manufacturer’s instructions. Briefly, seeds were frozen in liquid nitrogen and homogenized in a sample extraction buffer. After centrifugation at 3,000 × *g* for 20 min, the supernatant was subjected to ELISA using microtiter plates pre-coated with a GA, IAA, or ABA specific antibody, horseradish peroxidase-conjugated secondary antibody, and 3,3′,5,5′-tetramethylbenzidine chemiluminescence substrate. The absorbance was measured at a 450 nm wavelength using a microplate reader (Molecular Devices). The contents of lignin, cellulose, and hemicellulose of the lignocellulosic structure in the seed were measured using spectrophotometry according to Hussain et al. (2020). The absorbance value was read at 280, 520, and 620 nm wavelengths, and the content was calculated according to each formula, respectively.

The contents of nutritional substances, including soluble sugar, protein, starch, and fatty acid, were determined to investigate their correlation with germination traits. According to the anthrone method, soluble sugar and starch in the homogenized sample were hydrolyzed to glucose and subjected to an assay (Khan et al., 2000) and the anthrone-sulfuric acid method (Fernandes et al., 2012), respectively. Glucose was used as a standard for both assays, and anthracene was employed as the color reagent. Total proteins were extracted by the tissue homogenate method using an ice-cold PBS buffer. Protein in the supernatant after centrifugation was quantified following the Bradford method (Bradford, 1976). Total fatty acids were extracted from the samples from a freeze-dried powder following the method as described in Laffargue et al. (2007) and esterified to fatty acid methyl esters (FAMEs) according to the ISO-5509 standard. Gas chromatography/mass spectrometry analysis of FAMEs were performed following the procedures previously described (Laffargue et al., 2007).

### Germination

Undamaged and mature seeds were incubated in a Petri dish with filter paper wet and watered daily as necessary with deionized water. Each dish consisted of 25 seeds randomly selected from each sample. A total of 119 dishes were used for germination test. Petri dishes were put in an incubator under conditions that were 14/10 h light/dark cycle, and 30 ± 0.5°C during the day and 10 ± 0.5°C at night. Seeds were observed daily, and a radicle that emerged at least 1 mm long was considered as successful germination. Germinated seeds were removed from the Petri dish after counting. Positions of Petri dishes were changed randomly every 3 days. Seed germination rate, T_0_, T_50_, and the germination index (GI) were calculated for each population. Seed germination rate refers to the final germination percentage. T_0_ means the duration from the beginning of incubation to the first germination, while T_50_ indicates the period between the beginning of incubation and 50% of the seed germination rate (Ranal and Santana, 2006). GI is calculated using the formula: GI = Σ(Gt/Dt), where Gt represents the number of seeds that germinated on day t (Dt) (Ranal and Santana, 2006).

### Climatic Data

Climatic data for each sample location were downloaded from the China Meteorological Data Service Center^1^. Averages of annual temperature and precipitation from 2000 to 2018 were used in the correlation analysis between climatic variables and seed traits, based on data availability.

### Statistical Analysis

In this study, we determined four germination traits, four morphology traits, four phytohormone traits, three structural traits and nine nutritional traits in seeds of *A. artemisiifolia*. The coefficient of variation (CV, the ratio of the standard deviation to the mean) and Max/Min (ratio of maximum value to minimum value) were first calculated to depict the intraspecific variation in each trait. One-way analysis of variances were also applied to determine the intraspecies variation among populations with a significance level of *P* = 0.05. Then, principal component analyses (PCA) were performed on traits related to seed morphology (PCA_M_), phytohormone (PCA_P_), structural material (PCA_S_) and nutrient (PCA_N_) to reduce collinearity and dimensionality (Xiao et al., 2019). The scores of the PCA (axes 1 and 2), which were linear combinations of original traits, were used as representative variables (Xiao et al., 2019) and used for the following analyses.

To determine the impacts of latitude/longitude on *A. artemisiifolia* seed traits. we performed linear regressions to explore the relationships of seed germination traits (i.e., T_0_, T_50_, germination, and GI) and representative PCA variables with latitude/longitude. To explore how climate variables (i.e., mean annual temperature [MAT] and mean annual precipitation [MAP]) changed with latitude/longitude and whether there were differences in slope among the relationships, standardized major axis regressions were performed among above variables. Moreover, multiple linear regressions analyses were performed to analyze the relationships between seed germination traits and representative PCA variables. The full model was simplified using a stepwise model selection routine, and the most parsimonious model was selected based on the lowest Akaike information criterion (AIC) values. The normality and homogeneity of variances of the model residuals were checked using Shapiro-Wilk test and Bartlett test, respectively. Data were transformed to improve normality when necessary.

Structural equation modeling (SEM) was used to explore the direct and indirect effects of climatic variables on seed germination Index (GI). We assume that the climate directly influences the seed germination performance (e.g., GI) of *A. artemisiifolia* and indirectly *via* affecting seed intrinsic attributes. Noted that only GI was incorporated in SEM as it was a comprehensive indicator representing seed germination (Ranal and Santana, 2006). Climatic variables (MAT, MAP) and representative PCA variables that were most relevant with both latitude and GI were incorporated in the model. SEM analysis was performed with the maximum likelihood estimation method and the comparative fit index (CFI) was used to evaluate the goodness of fit. The CFI > 0.95 suggest the model was a good fit and an acceptable model. All the analyses were performed with MASS (Venables and Ripley, 2002), vegan (Oksanen et al., 2019), smatr (Warton et al., 2012) and lavaan packages (Rosseel, 2012) in R version 3.5.2 (R Core Team, 2018).

## Results

### Variation in Seed Traits and Climate Along Latitudes and Longitudes

**FIGURE 1:**
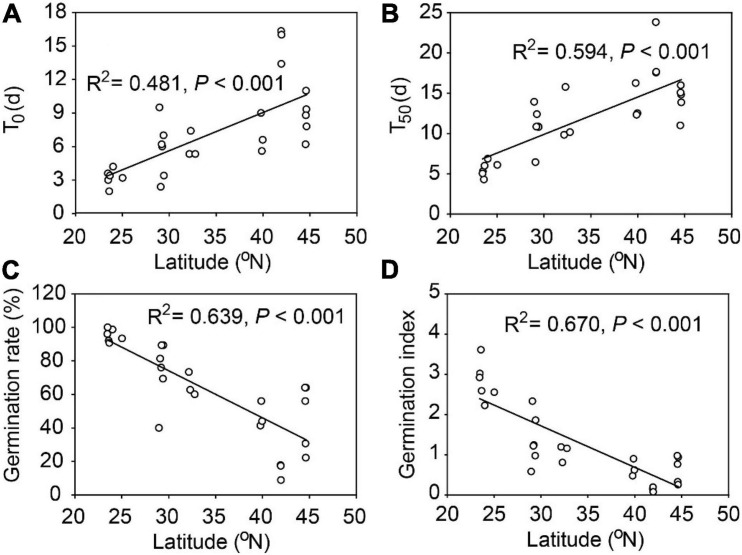
Seed germination traits for 26 populations of *Ambrosia artemisiifolia* along 23° latitudinal range in China. **(A)** T_0_, number of days of the first germination from the beginning of germination test; **(B)** T_50_, number of days required to reach 50% of final germination rate; **(C)** seed germination rate; **(D)** germination index (GI).

There were substantial variations in seed traits among the 26 populations of *A. artemisiifolia*, with exceptions of IAA, GA, the GA: ABA ratio, hemicellulose, and cellulose (Table 2). Among all the traits, seed germination characteristics had the highest Max/Min and CV values (Table 2) and had significant relationships with latitude (Figure 1) and longitude (Supplementary Figure 2). Specifically, the average time of T_0_ and T_50_ was prolonged by 3.41 days and 4.63 days, respectively, for every 10° increase in latitude (Figures 1A,B), however, the average germination rate and germination index were decreased by 28.1% and 1.03, respectively, for every 10° increase in latitude (Figures 1C,D). The average time of T_0_ (R^2^ = 0.377, *P* < 0.001) and T_50_ (R^2^ = 0.480, *P* < 0.001) was also significantly delayed with increasing longitude, while the seed germination rate (R^2^ = 0.494, *P* < 0.001) and germination index (R^2^ = 0.572, *P* < 0.001) had significantly negative relationships with longitude (Supplementary Figure 2). Note due to the sampling sites were highly correlated in latitude and longitude coincidentally (correlation coefficient: 0.95, *P* < 0.001), in text we only showed figures for the effects of latitudes and those for the longitudes were presented in Supplementary Figures.

**TABLE 2:** Seed trait variation of *Ambrosia artemisiifolia* from 26 populations in China.

**Seed traits**	**Maximum**	**Minimum**	**Max/Min**	**CV%**	**df**	**F**	***P***
**Germinational traits**							
Germination rate	100.00	7.00	14.29	45.31	25	**15.17**	** < 0.001**
Germination index	3.98	0.03	132.67	78.53	25	**20.47**	** < 0.001**
T_0_ (d)	30.00	2.00	15.00	70.17	25	**5.14**	** < 0.001**
T_50_ (d)	30.00	3.50	8.57	50.25	25	**6.22**	** < 0.001**
**Morphological traits**							
Length (mm)	5.18	2.14	2.42	18.95	25	**17.38**	** < 0.001**
Width (mm)	2.82	1.41	1.99	16.24	25	**17.77**	** < 0.001**
Seed mass (mg)	893.20	117.80	7.58	44.78	25	**21.69**	** < 0.001**
Moisture content (%)	6.93	1.74	3.98	21.17	25	**9.08**	** < 0.001**
**Chemical traits**							
** Phytohormone**							
IAA (ng g^–1^)	141.56	39.62	3.57	31.48	25	0.58	0.9402
GA (ng g^–1^)	826.54	307.66	2.69	26.51	25	1.50	0.0852
ABA (ng g^–1^)	1079.99	305.65	3.53	31.38	25	**2.271**	**0.0026**
GA: ABA	2.15	0.33	6.51	42.06	25	0.979	0.5020
** Structural material**							
Lignin (mg g^–1^)	308.37	210.15	1.47	10.54	25	**1.83**	**0.0202**
Hemicellulose (mg g^–1^)	130.28	96.55	1.35	7.21	25	1.00	0.4730
Cellulose (mg g^–1^)	152.47	113.65	1.34	6.79	25	0.64	0.8701
**Nutrient**							
Soluble sugar (mg g^–1^)	30.09	2.92	10.29	35.87	25	**2.973**	** < 0.001**
Protein (mg g^–1^)	22.61	1.48	15.26	37.63	25	**4.60**	** < 0.001**
Starch (mg g^–1^)	16.92	3.43	4.93	30.77	25	**3.08**	** < 0.001**
Total fatty acids (mg g^–1^)	121.88	64.71	1.88	14.84	25	**4.63**	** < 0.001**
Palmitic acid (mg g^–1^)	8.01	4.31	1.86	13.68	25	**5.02**	** < 0.001**
Stearic acid (mg g^–1^)	3.46	1.65	2.10	14.21	25	**5.18**	** < 0.001**
Oleic acid (mg g^–1^)	12.15	2.48	4.91	30.82	25	**5.85**	** < 0.001**
Linoleic acid (mg g^–1^)	42.34	22.14	1.91	15.43	25	**4.88**	** < 0.001**
Other fatty acids (mg g^–1^)	61.65	32.58	1.89	15.00	25	**4.68**	** < 0.001**

*CV, coefficient of variation; Max/Min, maximum value divided by minimum value; T_0_, number of days of the first germination from the beginning of germination test; T_50_, number of days required to reach 50% of final germination rate. Values of *P* < 0.05 are in bold.*

**FIGURE 2:**
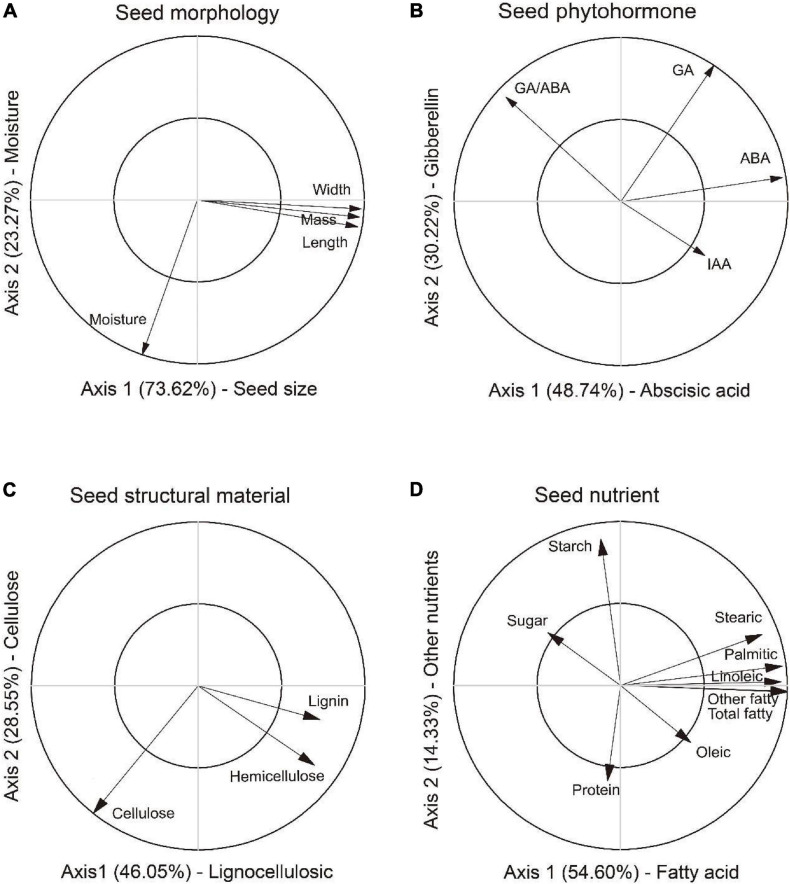
Principal component analysis for variables in morphology (PCA_M_), phytohormone (PCA_P_), structural material (PCA_S_) and nutrients (PCA_N_) in seed of *Ambrosia artemisiifolia*. The first two axes in PCA_M_ **(A)** were characterized as composite measures of “Seed size” and “Moisture,” respectively. The first two axes in PCA_P_ **(B)** were defined as composite measures of “Abscisic acid” and “Gibberellin,” respectively. In the PCA_S_ **(C)**, the first two axes were represented as composite measures of “Lingocellulosic” and “Cellulose,” respectively. In the PCA_N_ **(D)**, the first two axes were characterized as composite measures of “Fatty acid” and “Other nutrient,” respectively. PCA axes were used as representative variables for further analyses. Note only the significant relationships were shown in the figure.

In the PCA_M_ analysis, the first two axes explained 73.62 and 23.27% variation of the total variation, respectively (Figure 2A). The first axis positively correlated with length, width and mass, thus was defined as a composite measure of “Seed size.” The second axis was defined as a measure of “Moisture” as it correlated with moisture content. Greater values on the first and second axes suggested larger seed and higher moisture content in seed. The first two axes in the PCA_P_ analysis explained 48.74 and 30.22% of the total variation, respectively (Figure 2B). The first axis was defined as “Abscisic acid” as it was positively related to abscisic acid and ratios of gibberellin to abscisic acid. The second axis was defined as “Gibberellin” as it reflected the trade-off between GA and IAA. Greater values on the first and second axes means higher inhibitory and stimulation phytohormone contents in seed. In the PCA_S_ analysis, the first two axes explained 46.05% and 8.55% variation of the total variation, respectively (Figure 2C). The first axis reflected the trade-off among lignin, hemicellulose and cellulose, thus was defined as a measure of “Lingocellulosic.” The second axis were defined as a measure of “Cellulose” as it most relevant to cellulose and hemicellulose. Greater values on the first and second axes suggested higher contents in lignin and cellulose in seed. The first two axes in the PCA_N_ analysis explained 54.60 and 14.33% of the total variation, respectively (Figure 2D). The first axis was defined as “Fatty acid” as it was most relevant to composition of fatty acids. The second axis was defined as “Other nutrient” as it reflected the trade-off between sugar, starch and protein. Greater values on the first and second axes meant higher content in fatty acids and other carbohydrate in seed.

**FIGURE 3:**
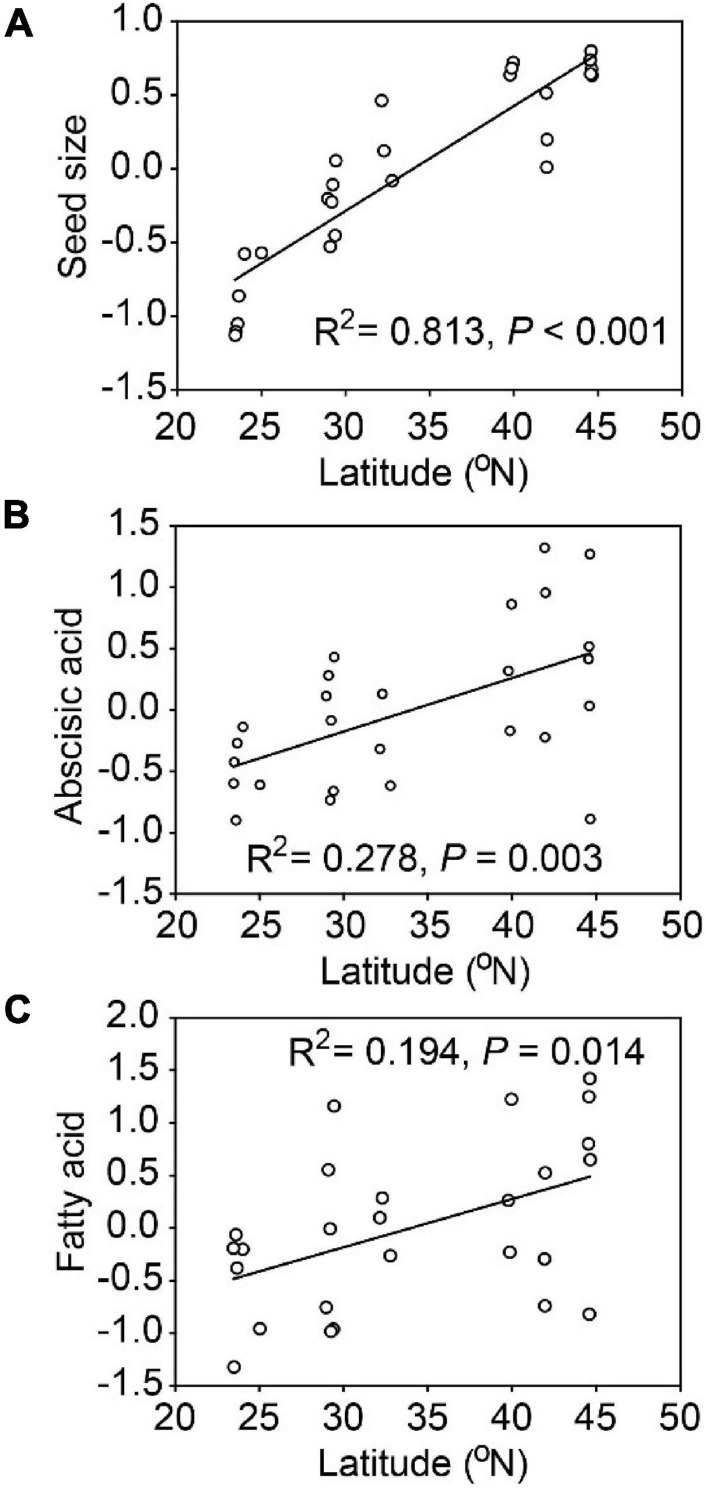
Relationships between latitude and representative variables (PCA axes) for *Ambrosia artemisiifolia*. The representative variables included seed size **(A)**, abscisic acid **(B)**, fatty acid **(C)**. Each point represents the mean value of all samples in a population (26 populations).

Seed size, abscisic acid and fatty acid were positively and significantly related to latitude (Figure 3). Populations of *A. artemisiifolia* at higher latitude tended to produce larger seeds, with higher phytohormone contents related to dormancy and greater nutrient concentrations in fatty acids. Similarly, seed size (R^2^ = 0.745, *P* < 0.001), abscisic acid (R^2^ = 0.195, *P* = 0.014) and fatty acid (R^2^ = 0.222, *P* = 0.008) were all positively and significantly related to longitude (Supplementary Figure 3). However, no significant relationships were observed among other representative PCA variables and the latitude/longitude.

MAT was significantly decreased with increasing latitude [slope: −0.80 (−0.84, −0.76)] and increasing longitude [slope: −0.86 (−0.77, −0.97)] (Supplementary Figures 4A,B), however, no differences were observed among these slopes (*P* = 0.206). MAP was also significantly and negatively associated with increasing latitude [slope: −57.03 (−49.56, −65.63)] and increasing longitude [slope: −61.81 (−49.65, −76.95)] (Supplementary Figures 4C,D), however, no differences were observed among these slopes (*P* = 0.531).

### Relationships Between Seed Germination Traits and Representative Variables

Multiple regressions revealed that seed germination traits had significant relationships with representative variables in seeds of *A. artemisiifolia*. Overall, seed size, abscisic acid and fatty acid were the most prominent predictors affecting seed germination, which explained more than 50% of variability (Table 3). Specifically, seed size was positively and significantly correlated with T_0_ and T_50_, but negatively correlated with germination rate and germination index. Abscisic acid exhibited significantly positive effects on T_0_ and T_50_, but exerted significantly and marginally negative effects on germination rate and GI, respectively. Fatty acid had significantly negative effects on T_0_ and T_50_, but had significantly and marginally positive effects on germination rate and GI, respectively.

**TABLE 3:** Effects of seed intrinsic traits on the seed germination traits of *Ambrosia artemisiifolia* from 26 populations.

**Germination Traits**	**Predictor**	**Estimate**	***t*-value**	**Prob (*t*)**	**AIC**	**R^2^**
T_0_	Seed size	3.133	2.883	0.008	55.804	0.507
	Abscisic acid	3.554	3.033	0.006		
	Fatty acid	−2.480	−2.836	0.010		
T_50_	Seed size	5.274	4.881	0.001	55.489	0.678
	Abscisic acid	3.379	3.074	0.006		
	Fatty acid	−2.484	−2.857	0.009		
Germination rate	Seed size	−25.855	−3.592	0.002	154.92	0.570
	Abscisic acid	−21.003	−2.823	0.010		
	Fatty acid	11.695	1.988	0.059		
GI	Seed size	−1.360	−6.809	< 0.001	−32.288	0.751
	Abscisic acid	−0.413	−2.034	0.054		
	Fatty acid	0.423	2.629	0.016		

### Direct and Indirect Effects of Climate on Seed Germination

**FIGURE 4:**
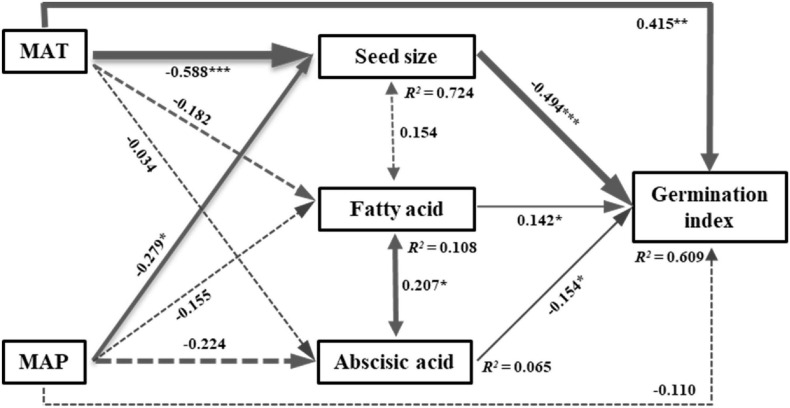
Structural equation model of the direct and indirect effects of climatic variables on seed germination index. Chi-squared = 3.307, df = 1, *P* = 0.069, CFI = 0.992. Numbers adjacent to the pathway are standardized path coefficients (*P < 0.05, **P < 0.01, and ***P < 0.001). Arrow widths are proportional to the standardized path coefficients. Portion of the variance explained by the model are indicated by R^2^ values. The solid and dash lines represent significant and non-significant effects, respectively. MAT, mean annual temperature; MAP, mean annual precipitation.

The SEM model including climate variables and representative PCA variables explained 63% of the variation in seed germination index (GI). The model fit well and acceptable, as indicated by the model fit parameters (Chi-squared = 3.307, df = 1, *P* = 0.069, CFI = 0.992) (Figure 4). Mean annual temperature (MAT) directly and indirectly affected the GI by negatively influencing seed size (Figure 4). Mean annual precipitation (MAP) had significant indirect effects on the GI by negatively impacting seed size (Figure 4). Thus, MAT contributed greater to the GI than MAP, regardless of indirect or direct effects. Fatty acid had significantly positive effects on the GI, while seed size and abscisic acid significantly negatively affected the GI (Figure 4). However, the path coefficient of seed size to the GI was greater than abscisic acid and fatty acid, indicating a greater contribution of seed size to the GI than other intrinsic traits (Figure 4).

## Discussion

We found strong but contrasting latitudinal trends in the seed traits and seed germination of *A. artemisiifolia* populations. Populations at lower latitudes demonstrate higher rates of seed germination, with smaller seed and lower abscisic acid and fatty acid concentrations. However, at higher latitudes, seed germination rate was lower, despite with larger seed and more nutrient resources. Our analyses reveal MAT, rather than seed intrinsic attributes, was the dominant driver of the variability in seed germination index. As latitudes and longitudes are highly correlated in our sample sites, the longitudinal trends of seed traits were similar to those of latitudinal trends. Our results indicate that *A. artemisiifolia* has adopted different germination strategies along latitudes/longitude in response to local climates.

This study found substantial seed trait variations in *A. artemisiifolia* among the 26 populations, and several seed traits exhibited latitudinal and longitudinal gradient patterns. Among seed traits, seed size is recognized as a fundamental ecological trait that influences the regeneration strategy of species, including seedling survival and distribution (Moles et al., 2007). In the present study, seed size increased from south to north along the latitudinal gradient, which was contrary to the results of studies on interspecific variations (Moles et al., 2007; Wu et al., 2018). However, our results are consistent with previous studies on our focal species, *A. artemisiifolia*. For example, van Boheemen et al. (2019) examined the relationship between seed mass and latitude among the plant’s native North American and introduced European and Australian populations, displaying that seed mass was larger at higher latitudes in Australian. Thus, interspecific and intraspecific seed trait variations may differ along latitude, likely reflecting difference between interspecific and intraspecific variations for plant adaptation to changing environments.

Generally, larger seeds have more resources which are beneficial to seedling survivorship and further development (Gómez, 2004; Moles and Westoby, 2004). In the present study, seeds from the high latitudes contained more fatty acids, which may contribute to seed size to tolerate cold weather. A previous study on sunflower, *Helianthus annuus*, reported that seeds with high linoleic acid concentration would germinate early at low temperature (González Belo et al., 2014). Meanwhile, the earlier and faster germination of smaller seeds in low latitudes may indicate the ability of seedlings to occupy an ecological niche to gain their dominance in the communities. Moreover, small seeds aid dispersal and accelerate range expansion (Muller-Landau et al., 2008; Tabassum and Leishman, 2018). Overall, these results suggest a high intraspecific variation in seed traits along a latitudinal gradient, indicating adaptative strategies of the invader to cope with the changing climates. Future studies combining field surveys, common garden experiments, and laboratory tests are warranted to provide clear evidence to support these assumptions.

Our findings on the variations in germination rate and timing between populations along latitudes/longitudes may result from the variation in seed hormones and nutrient contents. A previous study reported that germination-dormancy trade-off was the balance between inhibitory substance (ABA) and accelerative substance (GA, IAA) (Wang et al., 1999; Shu et al., 2016). The present study found increasing ABA as the latitude increased, indicating the roles of this plant hormone in regulating germination-dormancy under different environmental conditions. Relative to plants at low latitude, plants at high latitude are often affected by abiotic stresses such as frost, thus having shorter vegetation season. Therefore, dormancy has become a powerful tool/strategy to overcome these harsh conditions (Wagmann et al., 2012; Baskin and Baskin, 2014). In our study, a stronger dormancy in *A. artemisiifolia* at high latitude could increase the probability of survival and maintain population continuity. There was also a significant and positive correlation between latitude and fatty acids, suggesting fatty acids may be stored as potential energy resources at the later stages (Erbas et al., 2016). Previous studies reported higher temperature during seed development can greatly affect fatty acid by increasing the oleic/linoleic acid ratio in sunflower (Izquierdo et al., 2013), and seeds with high concentration of linoleic acid would show an advantage during germination at low temperature (González Belo et al., 2014). Mojzes et al. (2018) found seeds produced in different rainfall manipulation did not differ in offspring final germination rate, while seed mass was greater in less rainfall (drought) condition. Together, these results may reveal the physiological and biochemical mechanisms underlying seed germination variation along latitudes.

Seed traits and germination are affected by several abiotic and biotic factors (Baskin and Baskin, 2014; Saatkamp et al., 2019). We found that environmental variables in the local habitats along latitudes/longitude displayed a primary effect on seed germination patterns, indicated by the SEM analysis displaying the MAT significantly and positively affected seed germination. This is consistent with previous studies reporting the dominant role of temperature in germination performance and geographical patterns (Sang et al., 2011; Cai et al., 2016; Wu et al., 2018; Farooq et al., 2019). Therefore, it is possible that, because of climate change in the future, *A. artemisiifolia* could alter seed germination patterns through shifting seed intrinsic traits and then affect plant dispersal and establishment. In addition to the climate factors, the other abiotic and biotic environmental variables (e.g., soil nutrients, herbivores etc.) may also influence seed traits and germination progress, which needs further studies. But several studies have demonstrated that temperature and precipitation are the major factors affecting seed traits, plant performance, and distribution (Chamorro et al., 2018; Ramírez-Valiente et al., 2020; Zhou et al., 2020), as reported in the present study. Furthermore, the SEM analysis in this study indicated that the seed traits affected by MAT was stronger than MAP, which in line with previous study based on a worldwide meta-analysis (Moles et al., 2014). In this study, we sampled seeds from populations with low genetic difference (Li et al., 2019) but coincidentally their latitudes are highly correlated with longitudes, leading to the similar trends of their effects on seed traits. Future studies warrant testing their differences in affecting seed traits as changes in climates (temperature and precipitation) along latitude gradient may differ with those along longitude gradient.

In summary, we found substantial intraspecific variations in seed traits (i.e., seed mass, ABA, fatty acids, and germination) of the invasive *A. artemisiifolia* along a latitudinal gradient. Combined with the environmental variables, our results indicate their adaptive strategies in response to heterogeneous environmental conditions along latitudes/longitudes. Specifically, *A. artemisiifolia* seeds from lower latitudes germinated faster and had a higher germination rates, whereas seeds from higher latitudes showed contrasting patterns., Similar to *A. artemisiifolia*, many invasive plants reproduce by seeds and have invaded large geographic ranges. Thus, our findings may also expand to those invaders, deepening the understanding of seed adaptation strategies during the invasion process. We recommend that future studies on invasive plants across large geographic ranges (such as latitudes) should consider variations in seed traits and their ecological functions to provide a new perspective for understanding invasion success.

## Data Availability Statement

The original contributions presented in the study are included in the article/Supplementary Material, further inquiries can be directed to the corresponding author/s.

## Author Contributions

LZ, HY, and JD designed the experiments. LZ, KY, WY, and LC performed the experiments. LZ and HY analyzed the data. LZ, HY, and JD wrote the manuscript. All authors reviewed the manuscript.

## Conflict of Interest

The authors declare that the research was conducted in the absence of any commercial or financial relationships that could be construed as a potential conflict of interest.

## Footnotes

1 http://data.cma.cn/en/?r=data/weatherBk

## Supplementary Material

Supplementary Figure Location of 26 populations of *Ambrosia artemisiifolia* sampled along 23° latitudinal range in China. See Table 1 for site code.

Supplementary Figure 2 Seed germination traits for 26 populations of *Ambrosia artemisiifolia* along 20° longitudinal range in China. **(A)** T_0_, number of days of the first germination from the beginning of germination test; **(B)** T_50_, number of days required to reach 50% of final germination rate; **(C)** seed germination rate; **(D)** germination index (GI).

Supplementary Figure 3 Relationships between longitude and representative variables (PCA axes) for *Ambrosia artemisiifolia*. The representative variables included seed size **(A)**, abscisic acid **(B)**, fatty acid **(C)**. Each point represents the mean value of all samples in a population (26 populations).

Supplementary Figure 4 Relationships of latitude **(A,B)** and longitude **(C,D)** with mean annual temperature and mean annual precipitation in the sampling sites of *Ambrosia artemisiifolia*, respectively.
