# Supplementary material for: Latitudinal and Longitudinal Trends of Seed Traits Indicate Adaptive Strategies of an Invasive Plant
Source: Front Plant Sci. 2021 Jun 10;12:657813. doi: 10.3389/fpls.2021.657813 (PMC8222791; doi:10.3389/fpls.2021.657813)

**Supplementary Figure Captions**

**Fig. S1** Location of 26 populations of *Ambrosia artemisiifolia* sampled along 23° latitudinal range in China. See Table 1 for site code.

**Fig. S2** Seed germination traits for 26 populations of *Ambrosia artemisiifolia* along 20° longitudinal range in China. (A) T_0_, number of days of the first germination from the beginning of germination test; (B) T_50_, number of days required to reach 50% of final germination rate; (C) seed germination rate; (D) germination index (GI).

**Fig. S3** Relationships between longitude and representative variables (PCA axes) for *Ambrosia artemisiifolia*. The representative variables included seed size (A), abscisic acid (B), fatty acid (C). Each point represents the mean value of all samples in a population (26 populations).

**Fig. S4** Relationships of latitude (A-B) and longitude (C-D) with mean annual temperature and mean annual precipitation in the sampling sites of *Ambrosia artemisiifolia,* respectively.

**Fig. S1**


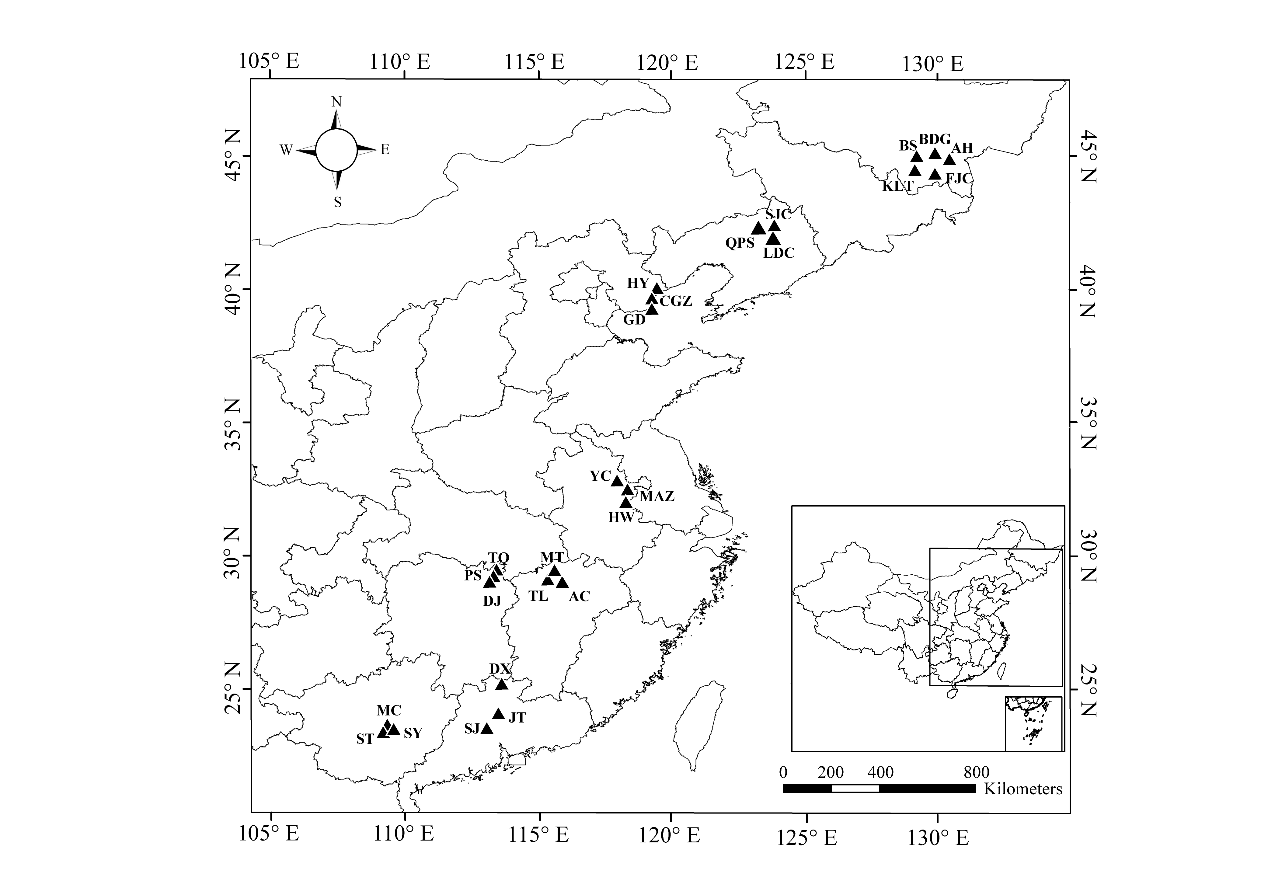


**Fig. S2**

**
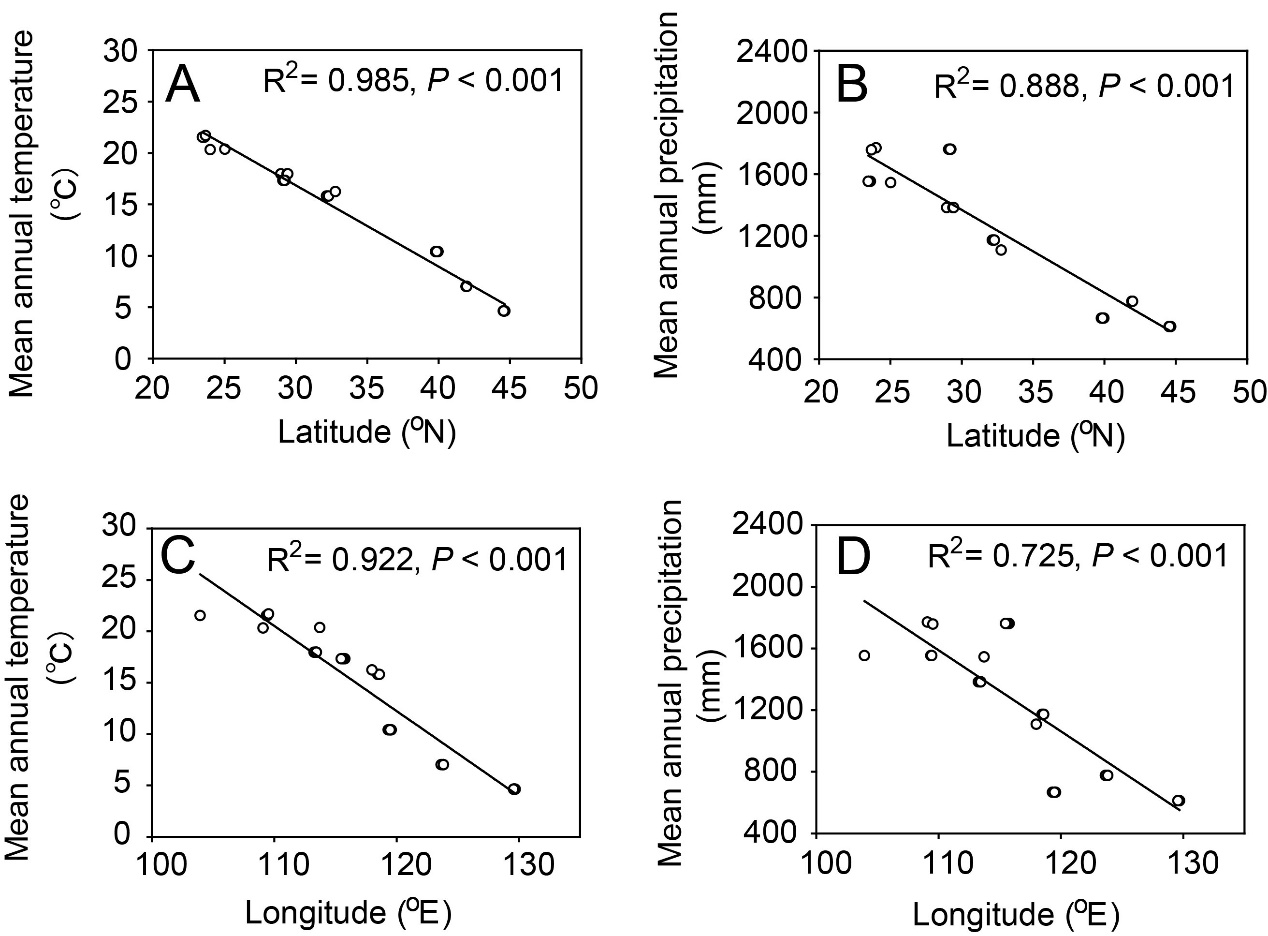
**

**Fig. S3**

**
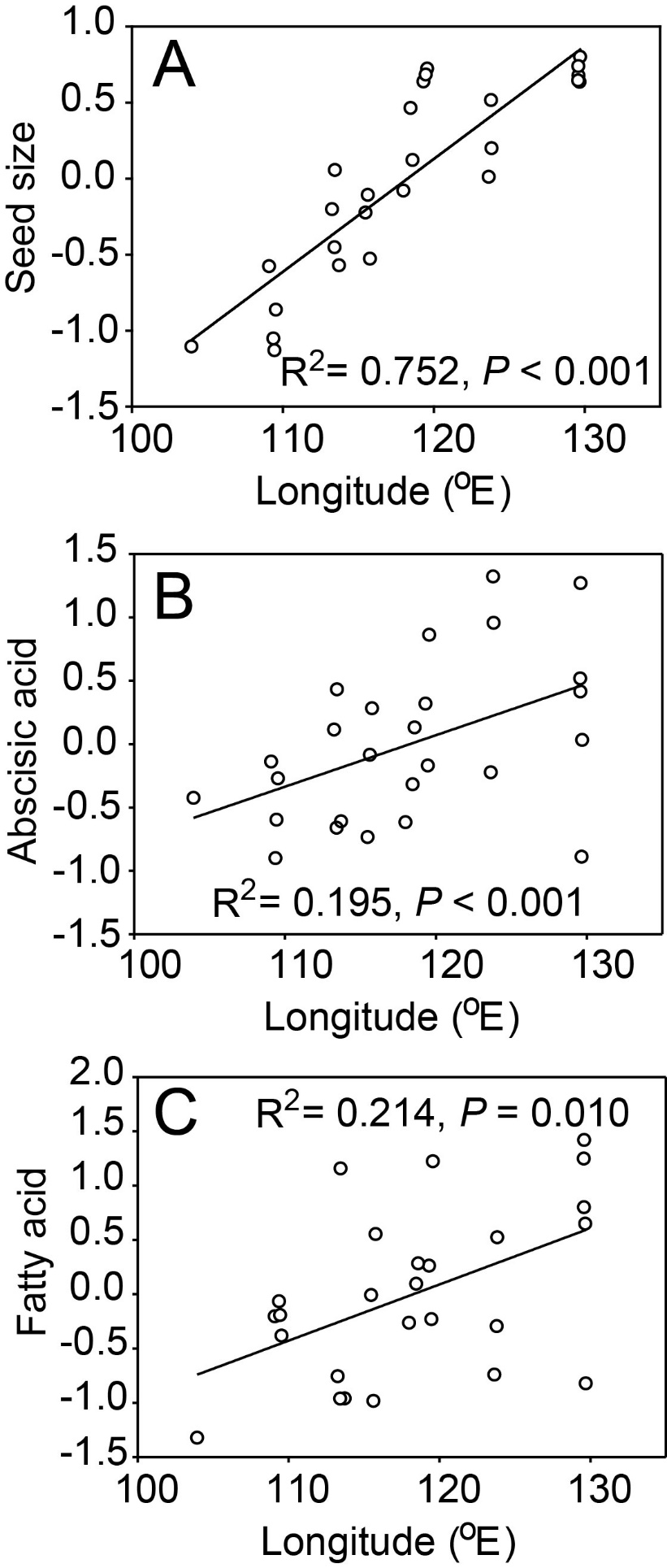
**

**Fig.S4**
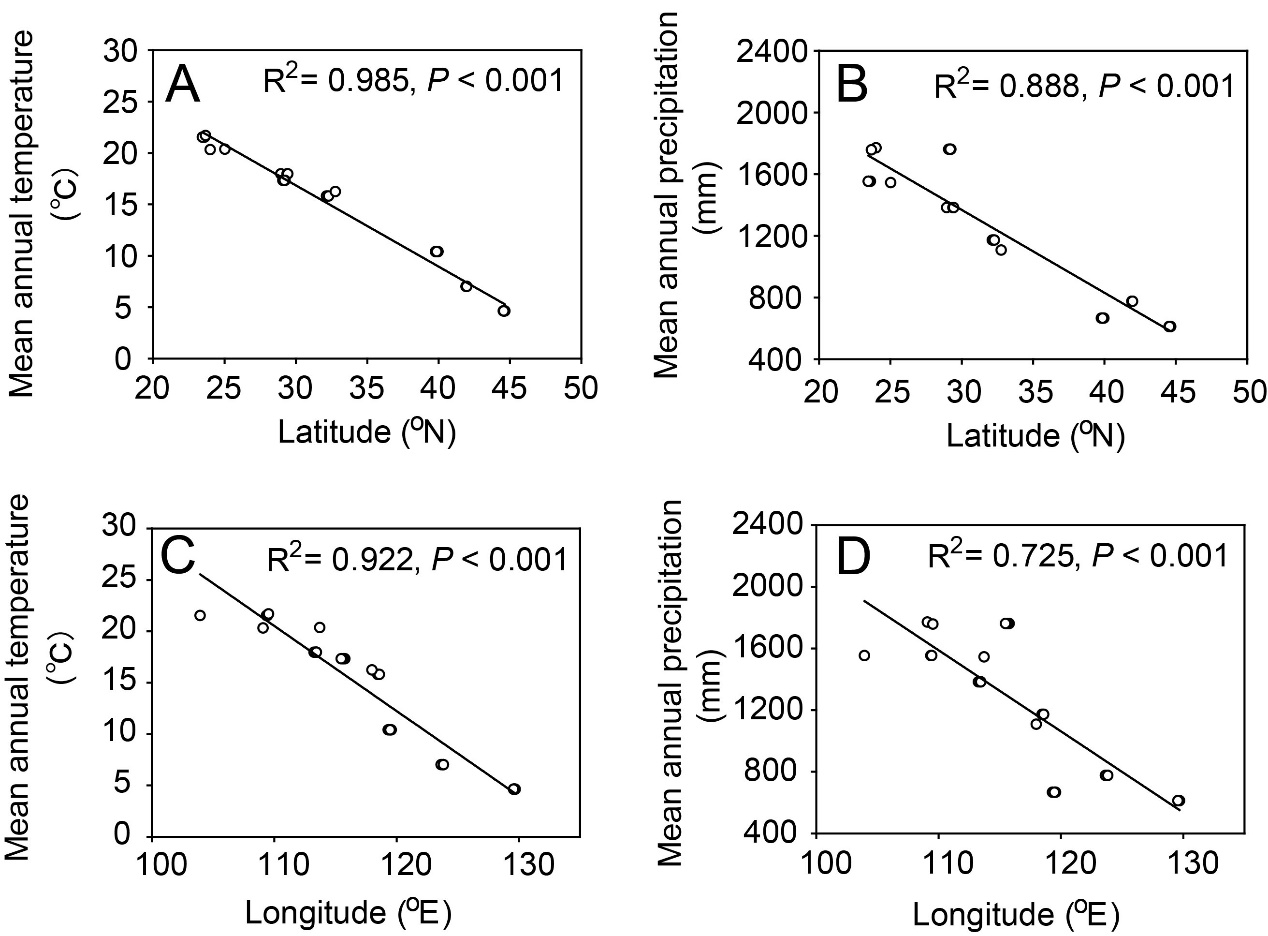

Supplement: Supplementary Figure — Location of 26 populations of Ambrosia artemisiifolia sampled along 23° latitudinal range in China. See Table 1 for site code. [file Data_Sheet_1.docx]
